# Off-label use and harmful potential of drugs in a NICU in Brazil: A descriptive study

**DOI:** 10.1186/s12887-016-0551-8

**Published:** 2016-01-21

**Authors:** Alcidésio Sales de Souza, Djanilson Barbosa dos Santos, Luís Carlos Rey, Marina Garruti Medeiros, Marta Gonçalves Vieira, Helena Lutéscia Luna Coelho

**Affiliations:** Pharmacy Department, Mother and Child Hospital of Brasilia, Brasília, Federal District Brazil; Doctoral Program in Development and Technological Innovation in Drugs, Federal University of Ceará, Fortaleza, Ceará Brazil; Mother and Child Hospital of Brasilia, SGAS, Av. L2 Sul, Quadra608, Módulo A, Asa Sul, Brasília, CEP 70203-900 DF Brazil; Postgraduate Programme in Pharmaceutical Sciences, Federal University of Ceará, Fortaleza, Ceará Brazil; Federal University of Recôncavo da Bahia, Santo Antônio de Jesus, Bahia Brazil; Mother and Child Health Department, Federal University of Ceará, Fortaleza, Ceará Brazil; Neonatology Department, Mother and Child Hospital of Brasilia, Brasília, Federal District Brazil

**Keywords:** Off-label use, Unlicensed medicines, Harmful excipients, High-alert medications, Neonate, Drug

## Abstract

**Background:**

Neonates admitted to neonatal intensive care units (NICU) are exposed to a wide variety of drugs, most without any data on safety and efficacy. Objective: To describe the drugs prescribed to different groups of neonates hospitalized in a NICU, and to analyze off-label use and harmful potential of drugs, in terms of the potential risks.

**Methods:**

This was a six-month retrospective cohort study of drug use in a NICU, with neonates who were inpatients for a period of over 24 hours, and using prescription data from electronic medical records. Drug information found in the package leaflets, in the British National Formulary for Children 2012–2013, and in the Thomson Micromedex database were compared. Drugs and excipients considered potentially harmful were evaluated according to the literature.

**Results:**

One hundred ninety-two neonates were included in the study, with a mean gestational age (GA) of 33.3 weeks (SD ± 4.3), 75.0 % were preterm, with an average of 18.8 days of hospitalization (SD ± 18.1), and a total of 3617 neonates-day. 3290 prescriptions were registered, on average 17.1 prescriptions/neonate (SD ± 17.9) and 8.8 drugs/neonate (SD ± 5.9). The number of prescriptions and drugs was higher in neonates with GA <31 weeks (*p* <0.05). Anti-infectives for systemic use, blood, alimentary tract and metabolism drug groups were more frequent, varying according to the GA. Neonates (99.5 %) were exposed to unlicensed drugs (UL) and off label use (OL), more frequently in GA <28 weeks (*p* <0.05). Most OL drugs used were indicated for newborns. 15 potentially harmful drugs were used in more than 70 % of the neonates, and most were OL; exposure to harmful excipients occurred in 91.6 % of the neonates, a percentage even higher when considering immature neonates.

**Conclusions:**

Immature neonates in a Brazilian NICU are exposed to a variety of OL, UL and potentially harmful drugs and excipients.

## Background

Lack of scientific evidence is one of the main problems involved in using medicine among neonates, especially those in critical care. Less mature neonates, with a gestational age below 32 weeks, and those with low birth weight (<2500 g) are frequently affected by apnea due to prematurity, neonatal encephalopathy, bronchopulmonary dysplasia and systemic infections. These problems explain the high use of drugs, which might reach 20 types concomitantly [1–3]. Almost 90 % of this use is off-label (OL) or unlicensed (UL), and should be considered experimental and be registered and followed carefully [3].

Medicine is an important innovation that has contributed towards improving neonate survival over recent decades, but adverse consequences from their use cannot always be foreseen, either in the short term or in the long term, as some effects are unpredictable [4]. Some serious, adverse events have been correlated with drug exposure, such as gastrointestinal complications relating to tolazoline, and the grey baby syndrome has been related to chloramphenicol [3]. Recently, ranitidine therapy was recognized as being associated with an increased risk of infections, necrotizing enterocolitis (NEC), and has a fatal outcome in very low birth weight (VLBW) newborns, but this and other drugs, such as domperidone, meropenem and cephalosporins, remain in use, despite the controversy [5–7]. The limited existing knowledge on pharmacokinetic development, pharmacodynamics and dose determination in relation to neonates, in association with the scarcity of clinical trials, may make it a complex task to select the right medicine and establish doses for treating neonates [4, 8].

There is no standard profile of drug use for neonates in critical care, other than some efforts towards standardization, such as establishing local or general guidelines [9–11] and publications like handbooks [12] and formularies [13] that are distributed worldwide. Almost all the medicine used in neonatal intensive care units (NICUs) is administered intravenously, and depending on gestational age and birth weight, the drug group most used is antibiotics, followed by drugs for use in the respiratory and nervous systems[2, 14–16].

In Brazil, drugs are authorized by the regulatory agency *Agência Nacional de Vigilância Sanitária* (ANVISA) but there is not a specific policy for registration of pediatric or neonatal drugs. There are 841 NICUs in Brazil, with a total of 8432 hospital beds, and national guidelines for prescriptions in neonatology are available only in cases of congenital infections, neonatal resuscitation, sepsis, pain and jaundice [17]. Studies in Brazil have shown a 5.5 to 12.6 % variation in UL use and 27.7 to 49.5 % in OL drug use, mostly relating to antibiotics, analgesics and drugs used in the digestive tract and metabolism [14, 18, 19], and the latter has been correlated with higher severity scores use [14, 20]. Furthermore, a preliminary study showed that neonates in NICUs were frequently exposed to potentially noxious excipients [21]. The present study aimed to describe the drugs prescribed to different groups of neonates hospitalized in a NICU, and to analyze off-label use and harmful potential of drugs, in terms of its potential risks.

## Methods

### Subjects and methods

This was a retrospective cohort study on drug use at a NICU with neonate inpatients, conducted over a six-month period in a tertiary-level facility [22].

The inclusion criteria were: neonates, aged ≤ 28 days, admitted in the NICU for more than 24 hours, who received pharmaceutical products to treat or prevent specific diseases and had completed electronic clinical records at the time of the study. Patients with incomplete clinical data, incomplete prescriptions or prescriptions containing only vaccines, blood products, parenteral nutrition, silver nitrate eye drops or intramuscular administration of phytomenadione in the delivery room, or intravenous hydration, were excluded.

Medicine information leaflets or the electronic leaflet database of the ANVISA were used to identify the excipients present in the formulations.

### Data collection

Prescription data and other information were obtained from the electronic medical records of the patient relating to his NICU stay. A specific form was used to collect patient’s information: gestational age (GA), birth weight (BW), BW adequacy, gender, date of birth, Apgar score at 1’ and 5’, diagnoses and data on the prescribed formulations: active ingredient, pharmaceutical company, pharmaceutical form, route of administration, daily dose, and beginning and end of treatment and the observed outcome (discharge, transfer or death). No data was recorded regarding intravenous hydration, vaccines, blood products, parenteral nutrition composition, use of silver nitrate eye drops or intramuscular administration of phytomenadione to patients in the delivery room. These last two drugs are of compulsory use in all neonates in the delivery room in Brazil, so they were not considered in the analysis [17].

### Classification of the drugs and excipients

Drugs were codified in accordance with the Anatomical Therapeutic Chemical classification [23] and were classified, as proposed by Neubert et al. [24], as follows: a) licensed (L)—drugs with registration issued by the ANVISA; b) unlicensed (UL)—drugs with no registration; or c) off-label (OL) use—use not detailed in the information leaflets, including therapeutic indication, patient age, strength (dosage), pharmaceutical form and route of administration categories. This classification was based on the information present in the information leaflets and on the websites http://www.anvisa.gov.br/datavisa/fila_bula/index.asp [25] and http://www.bulas.med.br/ [26].

Drugs were also analyzed according to their recommendations for use in neonates based on the British National Formulary for Children 2012–2013 (BNFC) [13] and the Thomson Micromedex© database [27]. Harmful excipients (adverse reactions reported) were categorized in accordance with Lass et al. [28] and Souza Jr et al. [21]. The composition of prescription formulations were determined from the information leaflets of the medicine, identified on the ANVISA database.

### Data processing and statistical analysis

Study subjects were classified as extremely preterm (<28 weeks of GA; EPN), preterm (28–36 weeks of GA; PN) and term neonates (>37 weeks of GA; TN); PN were subdivided into: a) 28–30 weeks of GA, b) 31–33 weeks of GA and c) 34–37 weeks of GA [2].

The exposure variables analyzed were: unlicensed medicines (UL), off-label use (OL) and exposure to harmful excipients. Exposure to drugs and harmful excipients was expressed as the exposure rate (ER), in which the numerator was the number of neonates exposed, at least to the drug or excipient concerned, one or more times, and the denominator was the total number of neonate-days at risk of using a drug or an excipient. The ER made it possible to evaluate the exposure of newborn infants to each drug or excipient. All data were stored and analyzed in Excel for Windows (version 7) and in the Statistical Package for the Social Sciences (version 18), using simple descriptive analysis; ANOVA and nonparametric tests (Mann–Whitney U and Kruskal-Wallis) were used for quantitative variable distribution, and Pearson’s chi-square test for nominal variables classified according to GA, with a significance level of *p* <0.05.

This study was approved by the Research Ethics Committee of the Health Department of the Federal District, Brazil (approval No. 021/2012, of January 23, 2012). The informed consent was not applied because it was dispensed by the ethics committee. This approval was based on the Resolution of the National Health Council No. 466/12 regarding the procedures of an observational and retrospective study, in which privacy and confidentiality of clinical data and the subjects involved were assured [29].

## Results

### Characteristics of the neonates

Patients were selected from a list issued by the nursing staff, containing 407 newborns hospitalized during the period, 206 of which were not included in the study (90 were not found in the system, 44 only received intravenous hydration, 37 with incomplete data and 35 of age > 28 days). Thus, 201 neonates who were inpatients for over 24 hours, for whom medication prescriptions had been recorded in the electronic system, were included in the study. Of these, nine were subsequently excluded because their prescriptions only related to delivery room care. Thus, the studied sample was 192 neonates. Out of this total, 100 (52.1 %) were male, 133 (69.2 %) were born by cesarean delivery, 40 (20.8 %) had Apgar 5’ less than 7 and 128 (66.6 %) needed to be resuscitated. The mean gestational age was 33.3 ± 4.3 weeks, and the majority of them were PN (75.0 %). The mean birth weight was 1909.5 g ± 886.0; 39 (20.3 %) were small for their gestational age and 70 (36.5 %) had a very low birth weight (<1500 g). The mean length of hospital stay was 18.8 ± 18.1 days, corresponding to a total of 3617 neonate-days. There was no significant variation in the average length of hospital stay among the subgroups of GA (p > 0.05). The mortality rate was inversely proportional to the GA (p > 0.05) (Table 1).

**Table 1:** Characteristics and prescription profile of neonates admitted to a NICU in Brazil

	Extremely preterm neonates (EPN)	Preterm neonates (PN)			Term neonates (TN)		
	<28 weeks	28–30 weeks	31–33 weeks	34–36 weeks	≥37 weeks	Total (n)	*p-*value*
N	23	34	48	39	48	192	--
Male (%)	43.5	50.0	58.3	53.8	50.0	52.0	0.804^a^
Cesarean delivery (%)	43.5	67.6	79.2	79.5	64.6	69.2	0.019^a^
Discharge (%)	17.4	38.2	56.3	59.0	66.7	51.5	<0.001^a^
Transfer (%)	17.4	14.7	27.1	23.1	22.9	21.8	<0.001^a^
Deaths (%)	65.2	47.1	16.7	17.9	10.4	26.5	<0.001^a^
Survivors (%)	34.8	52.9	83.3	82.1	89.6	73.4	<0.001^a^
APGAR 1’ (mean, SD)	6.0 ± 2.7	5.9 ± 2.0	6.5 ± 1.8	7.0 ± 1.6	6.8 ± 2.0	6.5 ± 2.0	0.139 ^b^
APGAR 5’ (mean, SD)	7.7 ± 2.2	7.9 ± 1.3	8.1 ± 1.1	8.5 ± 1.0	8.2 ± 1.3	8.1 ± 1.3	0.100^b^
Resuscitation (%)	69.6	79.4	68.8	64.1	56.3	66.6	0.275^a^
Birth weight (mean, SD- g)	804.0 ± 164.0	1078.4 ± 268.6	1681.4 ± 276.1	2244.5 ± 594.1	2983.7 ± 585.6	1909.5 ± 886.0	<0.001^c^
NICU stay (days)	24.0 ± 25.1	24.2 ± 19.5	14.7 ± 13.1	18.0 ± 15.0	17.3 ± 19.3	18.8 ± 18.2	0.203^b^
Neonate-days	552	824	706	705	730	3617	--
Prescriptions (mean, SD)	23.6 ± 24.4	22.5 ± 19.2	13.3 ± 12.3	16.6 ± 15.7	14.1 ± 18.6	17.0 ± 17.9	0.019^b^
Drugs (mean, SD)	11.5 ± 6.0	11.9 ± 5.3	6.9 ± 4.9	9.0 ± 5.8	7.1 ± 6.9	8.8 ± 6.1	<0.001^c^
Drugs/prescription (mean, SD)	4.1 ± 1.5	4.2 ± 1.4	2.8 ± 1.4	3.8 ± 1.8	3.0 ± 2.0	3.4 ± 1.7	<0.001^c^
>6 drugs (%)	73.9	85.3	41.7	53.8	37.5	54.7	<0.001^a^
Exposure to UL (%)	26.1	20.5	10.4	2.6	8.3	11.9	0.030^a^
Exposure to OL (%)	100	100	100	100	97.9	99.5	0.555^a^
UL and OL/prescription (mean, SD)	3.6 ± 1.3	3.5 ± 1.3	2.5 ± 1.2	3.3 ± 1.5	2.5 ± 1.8	3.0 ± 1.5	<0.001^c^
Exposure to high-alert medications (%)	82.6	97.0	60.4	69.2	64.5	72.4	0.002^a^
Number of high-alert medications (mean, SD)	2.6 ± 1.4	2.4 ± 1.5	2.1 ± 1.1	2.7 ± 1.5	2.7 ± 1.5	2.5 ± 1.4	0.561^b^
Exposure to harmful excipients (%)	91.3	100.0	93.7	92.3	83.3	91.6	0.101^a^
Formulations with harmful excipients (mean, SD)	4.5 ± 2.1	4.5 ± 2.0	3.2 ± 1.6	4.1 ± 1.7	3.8 ± 2.2	3.4 ± 2.1	0.001^b^

*SD* standard deviation

^a^Pearson’s chi-square

^b^Kruskal-Wallis test

^c^ANOVA

*p-value (<0.05)

The most frequent diagnosis was neonatal jaundice (115; 59.8 %) followed by respiratory distress (104; 54.1 %), sepsis (72; 37.5 %), anemia (56; 29.1 %) and hyaline membrane disease (HMD) (42; 21.8 %). The mean number of diagnoses varied depending on the GA and was higher among neonates with GA <31 weeks (*p* < 0.05) with a higher frequency of jaundice, sepsis and HMD, while congenital heart disease, seizures and hydrocephalus were observed more frequently among the TN.

### Prescribed drugs and formulations

During the study period, 3290 prescriptions were registered, with a median of 11 prescriptions/neonate (range: 1–103), with 103 formulations and 87 drugs. A total of 1725 drugs were prescribed, and the mean was 8.8 drugs/neonate (SD ± 6.1). The number of prescriptions, drugs and drugs/neonate were greater for neonates with GA < 31 weeks (*p* < 0.05) (Table 1).

According to the first level of the ATC classification, the highest risk to the neonates was exposure to systemic anti-infectives, with an exposure rate (ER) of 5.0 for every 100 neonates, followed by drugs that act on blood and blood-forming organs (ER 3.7), digestive tract and metabolism (ER 3.5), nervous system (ER 3.0), and respiratory system (ER 2.9). The ER varied with the GA and, thus, EPN were more exposed to drugs that act on the respiratory system (ER 4.2), whereas in the other GA groups, systemic anti-infectives were the drugs most frequently used, especially in the range of 31–33 weeks (ER 6.5). Neonates between 31 and 33 weeks had higher ER for drugs that act on the respiratory system (ER 5.5) and digestive tract (ER 4.4) than the other GA. Neonates with the GA greater than 34 weeks had a higher risk of exposure to drugs that act on the nervous system than the other GA groups. Preterm neonates with GA of 28–30 and 34–36 weeks were more exposed to drugs that act on blood and blood-forming organs: an ER of 4.4 and 3.6 for every 100 neonates, respectively.

According to the fifth level of the ATC classification, the most prescribed drugs were gentamicin (*n* = 110; ER = 3.0); ampicillin (*n* = 108; ER = 2.9); heparin (*n* = 97; ER = 2.6); phytomenadione (*n* = 95; ER = 2.6); aminophylline (*n* = 94; ER = 2.5); fentanyl (*n* = 92; ER = 2.5); multivitamins without minerals (*n* = 91; ER = 2.5); folinic acid (*n* = 83; ER = 2.2); dobutamine (*n* = 75; ER = 2.0); and vancomycin (*n* = 61; ER = 1.6). Neonates with a GA less than 34 weeks had higher incidence of aminophylline, whereas neonates with GA of 34-36 weeks and TN had a greater exposure to gentamicin and fentanyl, respectively (Table 2).

**Table 2:** Exposure rate (%) of drugs most prescribed of neonates admitted to NICUs in Brazil

GA < 28 weeks			GA 28–30 weeks			GA 31–33 weeks			GA 34–36 weeks			GA ≥ 37 weeks		
	*n*	*ER*		*n*	*ER*		*n*	*ER*		*n*	*ER*		*n*	*ER*
aminophylline	22	3.99	aminophylline	29	3.52	aminophylline	36	5.10	gentamicin	28	3.97	fentanyl	23	2.77
phytomenadione	19	3.44	phytomenadione	28	3.40	ampicillin	27	3.82	ampicillin	26	3.69	heparin	23	2.77
multivitamins without minerals^a^	18	3.26	multivitamins without minerals^a^	27	3.28	gentamicin	27	3.82	fentanyl	21	2.98	gentamicin	18	2.17
folinic acid	17	3.08	heparin	25	3.03	dobutamine	19	2.69	multivitamins without minerals^a^	20	2.84	ampicillin	17	2.05
ampicillin	16	2.90	folinic acid	24	2.91	phytomenadione	19	2.69	phytomenadione	19	2.70	metamizole	15	1.81
gentamicin	16	2.90	ampicillin	22	2.67	multivitamins^b^	18	2.55	folinic acid	18	2.55	dobutamine	15	1.81
fentanyl	15	2.72	gentamicin	21	2.55	multivitamins without minerals^a^	17	2.41	heparin	18	2.55	ranitidine	15	1.81
heparin	15	2.72	fentanyl	19	2.31	heparin	16	2.27	ranitidine	18	2.55	vancomycin	12	1.45
pulmonary surfactant	11	1.99	pulmonary surfactant	19	2.31	folinic acid	15	2.12	dobutamine	14	1.99	domperidone	11	1.33
meropenem	10	1.81	dobutamine	18	2.18	vancomycin	15	2.12	domperidone	13	1.84	alprostadil	10	1.20
vancomycin	10	1.81	domperidone	18	2.18	fentanyl	14	1.98	metamizole	12	1.70	phenobarbital	10	1.20
dobutamine	9	1.63	vancomycin	16	1.94	domperidone	11	1.56	multivitamins^b^	11	1.56	phytomenadione	10	1.20
amphotericin B	6	1.09	meropenem	14	1.70	amikacin	10	1.42	pulmonary surfactant	9	1.28	folinic acid	9	1.08
cefepime	6	1.09	multivitamins^b^	13	1.58	pulmonary surfactant	10	1.42	epinephrine	8	1.13	amikacin	9	1.08
domperidone	6	1.09	amikacin	8	0.97	ranitidine	8	1.13	furosemide	8	1.13	midazolam	9	1.08
teicoplanin	6	1.09	amphotericin B	8	0.97	cefepime	7	0.99	vancomycin	8	1.13	multivitamins without minerals^a^	9	1.08
epinephrine	5	0.91	cefepime	8	0.97	metamizole	6	0.85	aminophylline	7	0.99	furosemide	8	0.96
tricalcium phosphate	5	0.91	epinephrine	8	0.97	hydrocortisone	6	0.85	cefepime	7	0.99	epinephrine	6	0.72
multivitamins^b^	5	0.91	omeprazole	8	0.97	omeprazole	6	0.85	omeprazole	7	0.99	meropenem	6	0.72
ranitidine	5	0.91	tricalcium phosphate	7	0.85	epinephrine	5	0.71	amikacin	6	0.85	multivitamins^b^	6	0.72
ferrous sulfate	5	0.91	ranitidine	7	0.85	tricalcium phosphate	5	0.71	mineral oil	6	0.85	dexamethasone	5	0.68

*ER* exposure rate per 100 neonates

^a^vitamins A, B_2_, B_3_, B_5_, B_6_, C, D, E

^b^vitamins A, B_1_, B_2_, B_3_, B_5_, B_6_, B_8_, C, D_2_, E

### Unlicensed drugs, off-label use, potentially harmful drugs and harmful excipients

Among the 87 different drugs used on neonates, 15 (17.2 %) were unlicensed (UL), including magistral oral solutions (hydrocortisone acetate, folic acid, antimonium tartaricum, arginine, biotin, sodium benzoate, anhydrous caffeine, cyanocobalamin, tricalcium phosphate, furosemide, L-carnitine, pyridoxine, riboflavin and thiamine) and injectable alprostadil (500 mcg), which is imported. These formulations were prescribed to 23 neonates (12.0 %), of whom 19 received only one unlicensed drug. Out of the 1725 drugs analyzed from 3290 prescriptions, 38 (2.2 %) were classified as UL and 1687 (97.8 %) were licensed, of which 1358 were off-label use (OL). Among the OL categories, age was the most frequent, followed by dose, route of administration, dosage form and indication. In the OL category of age, neonates were most exposed to heparin (97; 50.5 %), fentanyl (92; 47.9 %) and multivitamins without minerals (91; 47.4 %) (Table 3).

**Table 3:** Brazilian license situation, indications for neonates and *off–label use* in neonates in Brazil

		MICROMEDEX			BNFC 2012–2013			
	n (%)	Indicated (%)	Not (%)	*NI* (%)	Indicated (%)	Not (%)	*NI* (%)	Most frequent drugs (neonates)
*Licensed*	329 (19.1)	78.7	21.3	--	75.4	24.6	--	domperidone (59)^a,c^, vancomycin (55), pulmonary surfactant (49), phytomenadione (47), furosemide (29), epinephrine (26)
*Unlicensed*	38 (2.2)	36.8	7.9	55.3	47.4	--	52.6	tricalciumphosphate (18)^b,d^, alprostadil (2), biotin (2), L-carnitine (2), riboflavin (2), thiamine (2)
*Off-label*	1358 (78.7)	64.8	22.9	12.3	90.6	3.0	6.4	
Age	869 (50.4)	55.6	33.0	11.4	87.8	2.8	9.4	heparin (97), fentanyl (92), multivitamins without minerals (91)^b^, aminophylline (87), ranitidine (53), metamizole (37)^a,d^, cefepime (31)^a,d^, omeprazole (29)^a^
Dose/frequency	345 (20.0)	82.9	1.7	15.4	98.8	1.2	--	gentamicin (110), ampicillin (108), amikacin (36), vancomycin (15), midazolam (3), ampicillin/sulbactam (5)
Age, pharmaceutical form	23 (1.3)	34.8	65.2	--	95.7	4.3	--	spironolactone (7), methadone (6), captopril (3), propranolol (2), sildenafil (2)
Age, route of administration	29 (1.7)	48.3	--	51.7	51.7	48.3	--	multivitamins without minerals (15)^b^, mineral oil (14)^b,c^
Route of administration	61 (3.5)	96.7	3.3	--	96.7	3.3	--	phytomenadione (59), nystatin (2)^a,c^
Age, form, route of administration	14 (0.8)	100.0	--	--	100.0	--	--	aminophylline (14)
Age, dose/frequency	8 (0.5)	100.0	--	--	100.0	--	--	aminophylline (8)
Indication, pharmaceutical form, route of administration	6 (0.3)	100.0	--	--	100.0	--	--	epinephrine (6)
Age, indication, pharmaceutical form	1 (0.1)	100.0	--	--	100.0	--	--	acetylsalicylic acid (1)^a^
Dose/frequency, pharmaceutical form	2 (0.1)	100.0	--	--	100.0	--	--	furosemide (1), levothyroxine (1)
Total	**1725 (100.0)**	**66.8**	**22.3**	**10.9**	**86.8**	**7.1**	**6.1**	

*NI* No information

^a^Not indicated by Micromedex

^b^Not found in Micromedex

^c^Not indicated by BNFC

^d^Not found in BNFC

Of all the 3290 prescriptions, 3145 (95.6 %) included OL drugs and 370 (11.2 %), UL drugs. Nearly all neonates were exposed to OL use (191; 99.5 %), and neonates of GA < 28 weeks had a higher frequency of exposure to prescriptions including UL or OL drugs (*p* <0.05) (Table 1).

Most drugs were indicated to neonates by both Micromedex and BNFC (66.8 % vs. 86.8 %), however Micromedex showed a greater number of drugs not indicated or without information than BNFC (33.2 % vs. 13.2 %). The most used drugs that were not indicated or not found in Micromedex were acetylsalicylic acid, mineral oil, multivitamins without minerals, metamizole, nystatin and omeprazole. According to BNFC, the drugs without information were cefepime, domperidone, metamizole, mineral oil, nystatin and tricalcium phosphate (Table 3).

Almost all neonates (91.6 %) were exposed to the following harmful excipients: methylparaben (ER 4.2 for every 100 neonates), propylparaben (ER 4.2), polysorbate 80 (ER 3.0), propylene glycol (ER 2.9), saccharin (ER 2.2), benzyl alcohol (ER 2.0) sodium benzoate (ER 1.8), ethanol (ER 1.8) and benzalkonium chloride (ER 0.4). There was no association between the number of exposed individuals and the GA (p > 0.05), although the average number of formulations containing harmful excipients was higher for EPN than for the other subgroups (*p* <0.05) (Table 1).

## Discussion

This paper presents a detailed analysis of drug use in a NICU in Brazil, and it is a pioneer study in the country in this aspect. While the number of observations of clinical records considered here is high, it has limitations in terms of the external validity. Primarily, because of the descriptive methodology of a wide range of newborn categories, diseases and drugs. Also, due to the retrospective character of the study, an important amount of clinical files were excluded due to incomplete data. Finally, it is based on a single NICU; even though a reference facility in the public health system in the Federal District, and covering a large population with a general profile of attendance. The conditions that led to hospitalization were similar to those seen in other NICUs in Brazil [14] and in other countries [16, 30], among which jaundice, sepsis, anemia and hyaline membrane disease were the most prevalent [31]. The length of hospital stay and the number of drugs were greater than found in other studies [16, 30], but lower than reported in preterm neonates admitted to an NICU in Germany (11.1 drugs/patient) [2], and in the USA (11.8 drugs/patient) [32].

According to ATC classification, systemic anti-infectives were the most prescribed therapeutic group, as observed in other settings [16, 30–32], with ampicillin, gentamicin and vancomycin predominating. Vancomycin use correlated more with empirical treatment of neonatal sepsis. Cefepime and meropenem use were also very frequent, despite not being considered routine second-choice antibiotics. In fact, issues have been raised in medical literature regarding their use in neonates [3]. Antibiotic use varies according to each NICU, and generally depends on criteria established by the local staff, as suggested by Liem et al [33] in a comparative study conducted in the Netherlands. Heparin and phytomenadione, respectively, were the drugs acting on blood and blood-forming organs that were most prescribed for TN and for those with GA < 31 weeks. Patients who received concomitant heparin and phytomenadione (*n* = 60) were greater than those receiving only heparin (*n* = 37) or phytomenadione (*n* = 35), thus suggesting that phytomenadione was used for treating heparin-induced thrombocytopenia during the hospital stay in the NICU. Heparin use (50 %) was greater than in USA and Estonia (47.0 % and 17.5 %, respectively) [15, 16]. Phytomenadione use was more frequent (49.5 %) than in Estonia (11.6 %) [16] and less frequent than in Germany (90.0 %) [2] and Ireland (81.1 %) [30], possibly because these last two analyses may have included phytomenadione administered intramuscularly in the delivery room, among other protocol differences.

Among the drugs acting on the digestive tract and metabolism, multivitamins without minerals were the most frequent, especially in neonates with a GA of less than 31 weeks. This prescribing practice is based on known vitamin deficiencies (A, D and E), which are common among premature babies. However, according to the BNFC 2012-2013, the toxic potential of these formulations should be considered when there is another source of supplementation, especially for vitamin A [13]. Domperidone, ranitidine and omeprazole were also widely used, for suspected gastric reflux disease, although such use is questioned by several authors who have taken the view that the evidence regarding the risks and benefits of these treatments is inconclusive[6, 7, 34]. Regarding cardiovascular drugs, dobutamine was the most prescribed (39.0 %), with a higher frequency than those found in studies conducted in Germany [2] and Estonia [16] (31.7 % and 12.2 %, respectively). Its use was greater in neonates with GA of 28–34 weeks; in most cases associated with hemodynamic instability due to septic shock.

In the group of drugs for respiratory tract disorders, aminophylline and pulmonary surfactant were the most prescribed, respectively, for apnea of prematurity and hyaline membrane disease, in preterm neonates. Aminophylline use was different to the recommendations adopted by other countries and by the WHO [35], where caffeine is considered to be a more effective and safer alternative [2, 16, 36]. In Brazil, oral and parenteral caffeine formulations are not commercialized, which increases the risk and hinders the access to imported formulations, or the use of magistral preparations [20]. Unlicensed drug use was associated with metabolic bone disease, inborn errors of metabolism and patent ductus arteriosus cases, and also involved magistral preparations or imported drugs. The prescription rate containing unlicensed medicines (12.0 %) was similar to that found in a study from Italy [23], but lower than that found in Estonia (22.0 %) [16], possibly due to different concepts for an “unlicensed drug”. The OL use, mainly with drugs that act on the digestive tract, and the nervous and respiratory system, was predominantly prescribed for the most immature neonates. The majority of the package leaflets had no information regarding the use in neonates, which may reflect an absence of clinical trials or outdated leaflets. Dose/frequency was the second most frequent category of OL use, providing a warning with regard to the risks of toxicity associated with the lack of reference pharmacokinetic studies on the target population. This was observed in relation to the antimicrobials ampicillin and gentamicin, for which the information leaflets recommended daily administration, whereas the prescriptions presented dosages and administration intervals varying according to the GA [13, 27], taking into account the renal immaturity of these patients [37]. It was also reported in relation to multivitamin use, in which twice the manufacturer’s recommended dose was widely prescribed (12 versus 6 drops, daily), containing tocopherol acetate, an excipient associated with E-Ferol syndrome [21].

Drugs for the digestive tract and metabolism, for the nervous system, and the systemic anti-infective group comprised of more than 50 % of off-label use, and were indicated for most neonates. The sources of information (information leaflet, Micromedex and BNFC) diverged in some cases. For example, domperidone was indicated for neonatal use by the manufacturer but not by other sources, which highlights the risk of extrapyramidal effects. Differences between BNFC and Micromedex were also observed: for example, Micromedex does not recommend omeprazole, ketamine and teicoplanin, while BNFC does. Another important observation is that no warnings regarding the maturity of the neonates were seen in the leaflets, except for gentamicin, vancomycin and multivitamins without minerals.

The analysis on the harmful potential of the drugs was based on their presence in the list published by ISMP [38]. Among the 15 high-alert medications identified, 93.3 % had off-label use, in particular drugs acting on the cardiovascular system and nervous system. Most of the neonates were exposed to these drugs, especially those of a lower GA. All high-alert medications were indicated for neonates, according to BNFC, but according to Micromedex, ketamine, methadone, metoprolol and norepinephrine have not been established as safe in this age group, and dopamine is a safer alternative for dobutamine [27]. Almost all the neonates, particularly the most immature of them, were exposed to formulations containing harmful excipients, especially parabens and polysorbate 80, for which the long-term effects are unknown [39]. Exposure to these excipients was mainly through oral formulations of drugs of questionable use, such as multivitamins and domperidone, as reported in a previous study [21]. The simultaneous or prolonged exposition to potentially harmful excipients can be an additional disease burden to neonates in critical conditions, which warns of the risk of toxicity due to concomitant and sometimes prolonged use of drugs containing the same excipients. In general, comparison of data on OL use and UL obtained in different contexts is difficult, primarily due to different study designs and methods. For this reason, we chose to use the consensus of Neubert et al. (2008) [24], to achieve comparable data and enable a broad discussion on the points raised. The limitations of this critical analysis reflects the controversial categories compared; OL use and use of harmful drugs and High Alert Medications per se are not necessarily wrong, and should be considered in light of the clinical needs and the existence of safer alternatives. But, even considering some degree of inconsistence of literature, exemplified by discrepancies between BNFC and Micromedex information, some practices identified in Brazil by this study, such as the use of silver nitrate eye drops, oral formulations multivitamins, domperidone, aminophylline, and ranitidine prescription, represents a known and avoidable risk for neonates. The use of silver nitrate in newborns in the delivery room is being questioned in literature, due to its limited efficacy, risk of chemical conjunctivitis and suffering on part of the babies, but in Brazil it remains obligatory [40]. The absence of a specific policy for pediatric/neonatology drug registration in Brazil reflects in some of the discrepancies between drug indication by the FDA and ANVISA, exemplified by the use of antiepileptics observed in a Brazilian study [41].

The authors consider this kind of study a contribution to the question about the needs of better drugs for neonates and of a more standardized use of them. Although depicting a profile of drug use in a NICU is important and useful, the heterogeneity and multiplicity of the clinical problems and stages of prematurity, allow different management among facilities, particularly in clinical situations where scientific evidence of drug efficacy is lacking. Studies that consider a single system (such as respiratory disease) or a single therapeutic group could result in a more reliable comparison. The context observed is characterized by drug use based on minimal scientific evidence, in terms of efficacy and safety, and with inappropriate formulations that therefore have questionable predictability. To improve this situation, it is necessary to fill the gaps in the fields of pharmacokinetics and pharmacodynamics on neonates, as well as to develop more adequate formulations and proper clinical trials on long-term safety, efficacy and neurodevelopmental outcomes of some of the commonly used off-label and unlicensed drugs related in this study. This situation is far from being solved, despite ongoing efforts. It might be possible to reduce its damage through consistent policies of the spreading of scientific evidence information, and by implementing appropriate conditions to apply this knowledge around the world.

## Conclusions

3290 prescriptions were analyzed, corresponding to 192 neonates and 3617 neonates-day. The number of prescriptions and drugs was higher in neonates with GA <31 weeks. Anti-infectives for systemic use, blood, alimentary tract, and metabolism drug groups were more frequent; varying according to the GA. More immature neonates presented a higher frequency of exposition to unlicensed drugs (UL) and off label use (OL). Almost all the neonates, particularly the most immature ones, were exposed to formulations containing harmful excipients, especially parabens and polysorbate 80. Exposure to these excipients was mainly through oral formulations of drugs of questionable use, such as multivitamins and domperidone.

The context issue is characterized by drug use based on minimal scientific evidence, in terms of efficacy and safety, and avoidable use of inappropriate formulations that therefore have questionable predictability.

## Abbreviations

ANVISA: Brazilian National Health Surveillance Agency
BNFC: British National Formulary for Children 2012-2013
BW: birth weight
EPN: extremely preterm neonate
GA: gestational age
HMD: hyaline membrane disease
ER: exposure rate
ISMP: Institute for Safe Medication Practices
NEC: necrotizing enterocolitis
NICU: neonatal intensive care unit
OL: Off-label use
PN: preterm neonate
TN: term neonates
UL: unlicensed
VLBW: very low birth weight
